# Notes and News

**Published:** 1898-01-15

**Authors:** 


### Notes and News.

(Collected and Communicated.)

A site costing £10,000 has been purchased at Knotty Ash by the local authorities for the erection of an infirmary for the aged poor of Liverpool.

The English hospital at Nice is to be enlarged in commemoration of the Queen's Jubilee. The Grand Duke Michael of Russia has consented to become a patron.

The new operating theatre has been opened at the Bolton Infirmary. The operating-room is 30 ft. long by 23 ft. wide, with bow windows on the north, south, and west sides of the building. The roof is circular, with glass all round; the walls are white and cream tiled. There is a vestibule, anæsthesing room, and doctor's consulting room. The cost is estimated at £1,260.

The new Cottage Hospital for Bishop Auckland has been fully decided upon, and Lady Eden will lay the foundation-stone some time this month. About £1,700 has been collected for it, a site opposite the workhouse has been secured from the Ecclesiastical Commissioners for £150, and three architects have submitted plans and offered to do the work on reasonable terms.

Once more the difficult question of a site for the new infirmary at Newcastle appears to be settled, and this time finally, it is to be hoped. The present arrangement provides practically for an exchange of land between the Corporation and the hospital committee. Some of the abandoned site of the old infirmary is to be handed over to the Corporation at a future period, whilst the new institution is to have its position on the Leazes.

A most beautiful window hast just been unveiled at the Scarborough Hospital. It is the Jubilee offering of the Children's Hospital League. The subject is Sir Joshua Reynolds' group of "Charity," and the figures of Faith and Hope, which form part of the celebrated window at New College Chapel, Oxford. The group of "Charity" is reproduced in the Prince of Wales's Hospital Stamp, from whence the idea of utilising it at Scarborough originated. No more beautiful design could have been chosen, and the choice in every way was a very appropriate one.

An anonymous donor has presented £500 to Guy's Hospital.

A number of cases of the plague continue to be recorded from Bombay. Out of 159 cases reported 126 deaths occurred.

Dr. J. Poland will lecture on deformities of bones after injury on Tuesday, 18th inst., at 5.30, at the City Orthopædic Hospital.

Four hundred guests, all representative of the best in German science, assembled to do honour to Professor Virchow at Berlin.

An epidemic of typhoid has broken out at the Hartlepool Workhouse. Upwards of twenty patients are reported, one amongst whom is the daughter of the workhouse master.

Influenza is again very prevalent in the metropolis. The present epidemic is noticeable from the liability to recurrence at very short intervals in the same patients.

The Lord Mayor, president of the Metropolitan Hospital, has consented to take the chair at the festival dinner to be held on Monday, April 18th next, at the Whitehall Rooms.

The Assistance Publique of Paris are gradually opening wards at their hospitals for the treatment of tuberculosis. At Lariboisiere two pavilions, containing 160 beds, are now in readiness.

The late Monsieur Philibert Dessaignes, Mayor of Champigny-en-Beaugé, has bequeathed £40,000 to the Loire and Cher Department for the establishment of a hospital for epileptics, the feeble-minded, the blind, and the deaf and the dumb.

We understand that it has been suggested, with regard to the prevalence of typhoid fever amongst the English troops in India, that Professor Wright, of Netley, should be engaged by the Indian Government to investigate the operation and the effects of prophylactic vaccination.

The Anti-Vivisection Society are keeping a vigilant eye upon the British Institute of Preventive Medicine, and the Home Secretary has received a letter from the Secretary of the Society asking him to refuse a licence for the new building on the Thames Embankment. The Home Secretary replied that no application for a licence had yet been made.

At a recent meeting at St. Petersburg the treatment of diphtheria was fully discussed with the light of a large experience of the epidemic throughout Russia. Last autumn the severest epidemic of diphtheria ever recorded there was raging at St. Petersburg, and the testimony from there and from all parts was highly in favour of the serum treatment as reducing the death-rate.

A cheque for £10,000 has been handed over to the Leicester Infirmary as a Jubilee endowment of the institution. The bazaar held on behalf of the Infirmary some time since, at which the Duchess of Rutland, the Countess of Warwick, and Mrs. de Lisle lent their assistance, produced £2,500, besides stimulating much interest in the infirmary, resulting in increased annual subscriptions.

